# Supplementary material for: Are carbon emissions trading and green financial instruments synergistic? -Comprehensive quantitative research based on content analysis
Source: PLoS One. 2024 Mar 7;19(3):e0298601. doi: 10.1371/journal.pone.0298601 (PMC10919601; doi:10.1371/journal.pone.0298601)
Supplement: S1 Data — (ZIP) [file pone.0298601.s001.zip › Data/地级市Z_F工作报告.zip!/║■─╧/╔█╤⌠╩╨/└┤╫╘╣1⁄2╓┌║┼í╛┬φ┐╦╩2╛▌═°í┐_2016.docx]

各位代表：

现在，我代表市人民政府，向大会作工作报告。请各位代表连同《邵阳市国民经济和社会发展“十三五”规划纲要（草案）》一并审查，并请各位政协委员和列席人员提出意见。

一、“十二五”及2015年工作回顾

过去五年，在省委、省政府和市委的坚强领导下，在市人大和市政协的监督支持下，全市上下主动适应新常态，全力应对经济下行，化解各类风险，克服多重困难，总体完成了“十二五”目标任务，为全面建成小康社会奠定了坚实基础。

过去五年，是综合实力大幅提升的五年。2011年到2015年，主要经济指标连年保持两位数增长，增幅高于全省平均水平，绝大多数指标实现翻番。GDP迈上1000亿元台阶，由728亿元增加到1390亿元（预计数，下同），年均增长11%；财政总收入突破100亿元，由51.9亿元增加到131亿元，增长1.5倍，年均增长20%；规模工业增加值由211亿元增加到520亿元，增长1.4倍，年均增长14.1%；固定资产投资由416亿元增加到1515亿元，增长2.7倍，年均增长30%；社会消费品零售总额由280亿元增加到746亿元，年均增长14.7%。在全省绩效考核中，邵阳市成为唯一连续三年评为先进的市州。2014年邵阳市荣获全省全面小康建设“经济发展奖”，是大湘西4个市州中唯一获奖地区。

过去五年，是经济结构更趋优化的五年。三次产业由24:40:36调整为21.7:36.7:41.6。新增3个过百亿元产业，产值过亿元企业由159家增加到550家，其中10亿元企业12家。新增省级工业集中区7个。高新技术产业产值达到730亿元，年均增长20%。农业产值和粮食总产实现五连增，其中粮食总产达到354万吨，列全省第一。建成油茶、奶业、药材等特色农业生产基地245万亩，创建省部级畜禽渔标准化示范场53个。农产品加工规模企业由258家增加到374家。服务业日益活跃，物流企业达到400多家。累计接待游客9507万人次，旅游收入达695亿元，占GDP比重由8%提高到14%。新增各类融资1190亿元，金融机构存款余额由946亿元增加到2048亿元，贷款余额由347亿元增加到902亿元，存贷比达到44%，提高7个百分点。

过去五年，是发展后劲显著增强的五年。怀邵衡铁路开工建设，娄邵铁路扩改竣工并开行高铁，沪昆高铁邵阳段通车，邵阳市已跨入“高铁时代”。洞新、怀通、娄新、邵坪4条高速公路相继通车，安邵、武靖高速公路加快建设，实现“县县通高速”。修通干线公路725公里、农村公路2800公里。武冈机场有望今年试飞。治理4座中型、1040座小型病险水库，建设城市防洪堤25.5公里，治理河道320公里。建成和在建风电项目总量居全省第一，光伏、生物质能发电加快推进。新建220千伏变电站5座，新建和改造110千伏变电站17座，邵阳市成为全省输变电枢纽城市。4G信号覆盖绝大部分行政村。

过去五年，是城乡面貌发生较大变化的五年。全市城市总体规划、县市城镇体系规划修编和村庄布局规划编制工作有序推进，中心城区控规覆盖率100%。城镇化率由32.8%提高到41%。中心城市建成区面积由50平方公里增加到70平方公里，人口由55万增加到70万，建成一批骨干道路和配套设施，管输天然气正式入邵，综合承载能力显著增强。成功创建省级文明城市和省级园林城市，正在全力创建国家卫生城市。各县城和中心城镇建设提速。深入开展农村环境卫生综合整治和“美丽乡村”建设，城乡环境卫生大为改观，新农村建设成效明显。

过去五年，是改革开放成效最大的五年。9大类154项重点改革扎实推进，农村综合、文化体制、医药卫生、财税金融、工商登记、投融资等各项改革全面实施，特别是对长期拖累邵阳市经济社会发展的国企、公路管理、城市管理、自供区等体制实行成功改制和改革。累计完成进出口总额34.6亿美元，年均增长31.3%；利用内资3125亿元，年均增长20.6%；利用外资6.8亿美元，年均增长17%。新增境外投资总额9.4亿美元，年均增长27.5%。邵阳市成功与澳大利亚新南威尔士州怀昂市结为友好城市。

过去五年，是群众得到更多实惠的五年。城镇居民人均可支配收入突破2万元大关，由11686元提高到21270元，农民人均纯收入接近万元关口，由3706元提高到8600元。城镇登记失业率控制在4.2%以内。养老、医疗保险基本实现全覆盖，被征地农民社会保险正式实施，城乡低保应保尽保，保障性安居工程加快推进，住房公积金覆盖面持续扩大。建成公办幼儿园135所、农村合格学校800所，中小学校基本配齐标准桌椅。文化事业蓬勃发展，文艺创作精品不断涌现。祁剧演员肖笑波荣获戏剧表演国家最高奖项梅花奖，填补了祁剧空白。群众体育活动蓬勃开展，成功承办了一系列国家级和省级赛事。医疗资源配置不断优化，公共卫生服务项目不断增多，完成四级医院扩改，新建各类医院36所，新增病床3191张。人口自然增长率控制在6.6‰以内。物价水平和安全生产总体平稳。

过去五年，是生态文明不断进步的五年。植树造林、公益林保护、退耕还林和石漠化治理等生态工程进展良好，森林覆盖率达61.03%，全市整体纳入国家生态文明建设先行示范区。耕地保护基本国策进一步落实，连续15年实现耕地占补平衡。城市生活垃圾无害化处理率达94.2%，城市污水处理率达86.5%。单位GDP能耗累计降低18%，化学需氧量、氨氮、二氧化硫和氮氧化物的削减率分别为6.6%、9.1%、9.5%和2%，全面完成“十二五”节能减排任务。

过去五年，是法治邵阳建设扎实推进的五年。自觉接受市人大法律监督、工作监督和市政协的民主监督，认真听取和采纳民主党派、工商联、无党派人士和人民团体的意见建议，累计办理人大代表建议1041件、政协提案2023件，办复率100%，满意率达99%以上。市本级许可项目和非行政许可项目精简率超过70%。制定并公布市县政府工作部门的权力清单、责任清单和负面清单。“12345”政府服务热线正式开通。市民法制学校建设经验全国推介。领导包案化解信访积案的做法列为湖南省十大法治事件。落实重大事项专家咨询、公示、听证等制度。电子政务、政府信息公开和市县乡三级政务服务体系不断完善。审计监督、廉政建设和反腐败工作取得较好成效，“三公”经费逐年下降，市本级2014年下降32%、2015年又下降10.5%。平安邵阳建设成效明显，社会大局和谐稳定，群众工作站经验全省推介。国防动员和双拥共建工作扎实开展，国家安全和人民防空事业得到加强，1216、1213人防工程竣工，驻邵解放军和武警部队在支持地方建设等方面发挥了重要作用。

工商、质监、移民、供销、市志、统计、档案、贸促、人事编制、民族宗教、外事侨务、食药监管、邮政通讯、气象水文等各项工作都取得新进步，工会、共青团、妇联、残联、工商联、社科联、红十字会等群团组织作用进一步发挥。

各位代表，2015年是“十二五”的收官之年，我们按照“三严三实”要求，创造性地开展工作，用优异成绩为“十二五”划上了圆满句号。一是坚持把项目建设作为稳增长的关键支撑。实施310个重点建设项目，完成投资612亿元，增长12.3%。二是推进三次产业持续发力。把湘商产业园建设作为“一号工程”，建设进度全省领先。继续实施工业“四百工程”，项目开工145个，竣工130个，招商150个，新增规模工业企业150家，新增数量居全省第二。新增省级农民合作示范社34家、家庭农场398家。现代服务业迈出新步伐，新型业态发展活跃。邵东获授国家电子商务进农村试点县，城步获授湖南省农村电子商务示范县，“洞口农业产业带”在中国惠农网上线运行，雪峰蜜桔、武冈卤菜网上电子交易额屡创新高。崀山景区创5A通过国家验收，上堡古国侗寨申遗工程加紧推进，蔡锷故居保护开发建设完成主体工程。新开业和新批筹27家金融机构，新增进入湖南省重点上市后备资源库的企业7家，17家企业在新三板、新四板等多层次资本市场挂牌。三是城乡建设呈现新气象。中心城区“最美十条街”陆续竣工，文化艺术中心主体建成，湘林豪廷酒店开业运营，市行政中心即将竣工，市体育中心建设进展顺利。城区主街干道清扫保洁全面市场化，环卫机械化作业率40%以上，市容管理更加规范，长效机制逐步构建。拆除存量违法建设47.7万平方米，增量为零。邵东廉桥镇、城步大寨村等一批镇村被评为全国重点镇、名镇名村和全省美丽乡镇、民族特色村寨。四是全面深化改革开放。市县政府机构改革、乡镇区划调整改革、公路管理体制改革、商事制度改革基本完成。公车改革有序推进。市属国有工交企业改革扫尾进展较快，煤炭企业和湘运公司改制基本完成。完成47个大中型水管单位体制改革和98个小水电自供区改革。县级公立医院综合改革全面实施。成功举办湘商大会、湘台经贸会等重大经贸活动。五是社会事业全面进步。专利申请1707件，专利授权994件。新建省级工程技术中心2个、省级公共科技服务平台2个、院士专家工作站2家。有效注册商标总量位居全省第二。幼师专科学校、市一中搬迁、湘郡铭志学校等20个教育发展重点项目进展顺利，邵阳学院、邵阳医专成功合并。市中心医院东院等一批医疗项目建设进展顺利。人口自然增长率7.2‰，符合政策生育率78.3%。完成“送戏下乡，演艺惠民”活动和“雅韵三湘”高雅艺术普及计划1025场，放映农村公益电影69750场。举办邵阳市第三届艺术节。完成1569个行政村广播“村村响”，10309个自然村广播电视“村村通”。举办第一届中国湖南（南山）六月六山歌节、第一届绥宁四月八姑娘节活动。六是着力推进民生改善。全面完成28个为民办实事项目。城镇登记失业率控制在4%以内。新建农村敬老院11所、幸福院262所、养老服务示范点50个。开工公租房项目293个、128万平方米，城市棚户区改造项目47个、174.6万平方米，国有工矿棚户区改造项目3个、17.3万平方米，改造农村危房23400户。建设大中型饮水工程350处，解决55.3万农村人口饮水不安全问题。深入开展命案侦破、打黑除恶、“秋冬会战”等专项行动，社会治安明显好转。未发生较大以上生产安全事故，被评为全省先进单位。

各位代表，五年弹指一挥间，我们积累了弥足珍贵的经验。总的一条，就是蹄疾步稳，稳中快进。一是积极主动对接国家大政方针，干了一些大事。争取并办好了武陵山片区发展、老工业基地改造、产城融合示范区以及兴永郴赣铁路、怀邵衡铁路、呼南高铁客专等一大批事关国计民生长远发展的大事。娄邵盆地高标准基本农田项目，投资50亿元，为邵阳市建国以来最大的国家投资农业项目。棚户区改造上级补助资金累计17.2亿元，国开行低息贷款141.8亿元。提前实施水电自供区改制，获得上级改造资金22亿元，二年全部完成农网改造。累计发行专项债券65亿元，有效解决建设资金瓶颈问题。国企改制省级挂账13亿元和支持资金5亿元。二是坚持项目第一抓手，干了一些实事。连续实施“项目建设年”活动，五年累计实施重点项目672个，总投资4800亿元，实际完成投资1968亿元，陆续开工和竣工了一大批打基础、利长远的重大项目。组建国资投、交通投、社会投、园区投、水利投等融资平台，引进一批股份制商业银行，推进农信社改制，筹建村镇银行，发展融资性担保机构，为项目建设提供持续资金保障。破解征拆难题，实行房屋拆迁集中安置改革，推行“三榜公示、两级审核、经费包干”的阳光征拆模式。加强项目管理，有效规范了项目指挥部职能职责和内部管理，项目管理实现制度化、规范化。三是敢于担当，干了一些难事。我们对前进道路上的问题不掩饰、不回避，凝聚各方智慧，以无畏的勇气，迎难而上，啃下了一块块过去想啃而没有啃下的“硬骨头”。在全省率先关闭落后小煤矿93家，被誉为“邵阳模式”，经验在全国推介。坚决实施并提前完成国企改革三年攻坚计划，实现了完成任务多、筹措资金多、化解矛盾多和总体效果好的“三多一好”，经验在全省推介。平稳、有序、成功实施了中心城区“限摩限电”，极大地改善了交通和治安状况。持续强力推进中心城区综合整治，坚定创建国家卫生城市，高标准改造小街小巷367条，新建和改造农贸市场42个、公厕569座，配套完善地下管网791公里，坚决拆除违法建筑，城市形象和品位大幅提升，让邵阳彻底告别了“脏、乱、差”的历史。四是突出民生至上，干了一些好事。开展水、电、路、气、房、环境改善“六到农家”，解决了238万农村人口饮水不安全问题，农网改造即将全部完成，农村公路通达工程100%到村，推广户用沼气池1.6万口，每年改造农村危房1万户以上，城乡环境综合整治初见成效。五年共实现68万贫困人口脱贫，贫困地区群众人均纯收入每年增幅高于全市平均水平。群众幸福指数持续攀升，综治民调全省排名逐年进位。

各位代表，创造的物质来之不易，培育的精神影响深远。这是省委、省政府和市委正确领导的结果，是人大、市政协及社会各界支持参与的结果，是全市人民共同努力的结果。在此，我代表市政府，向全市人民，向人大代表、政协委员，向各民主党派、工商联、无党派人士和人民团体，向驻邵人民解放军、武警官兵和公安政法干警，向部省属驻邵各单位，向关心支持邵阳发展的各界朋友，致以崇高的敬意和衷心的感谢！

各位代表，成绩属于过去，问题是前进方向。邵阳市经济社会发展中还存在不少矛盾和问题，主要体现在：实现全面小康社会的程度不高，经济总量、人均均量不大，运行质量不高，扶贫任务艰巨；供给侧和需求侧的结构性矛盾比较突出，第一产业大而不强，第二、三产业比重不高；基层基础不牢，安全生产、综合治理和计划生育等底线工作还很薄弱；干部驾驭和应对经济下行和发展的观念、责任感和能力需要增强，公共服务水平需要提高。我们将高度重视这些问题，采取有力措施加以解决。

二、“十三五”的目标任务

“十三五”时期，党中央决定实施“四个全面”战略布局，坚持“五位一体”的发展思路，全力推进和落实创新、协调、绿色、开放、共享五大发展理念，奋力冲刺国内生产总值和城乡居民人均收入比2010年翻一番的倍增目标，为实现“两个一百年”奋斗目标和中华民族伟大复兴中国梦打下坚实基础。这是邵阳市未来发展的根本遵循和宏观机遇。邵阳市仍将处于人均GDP2000—10000美元的高速增长期，发展潜力巨大，内生动能强劲。省委、省政府确定的“一核三极四带多点”的战略布局，必将重塑邵阳市处于湖南东部长株潭到西部大湘西过渡带、北部洞庭湖到南部湘南结合部的“一带一部”区位新格局。这是邵阳市未来发展的阶段性特征和中观机遇。国家、省全面实施去产能、去库存、去杠杆、降成本、补短板五大任务，将大大促进实体经济发展和基础设施建设。这是邵阳市未来发展的基本要求和微观机遇。当然，我们也面临国家转方式调结构、经济下行、“马太效应”等严重挑战。但是，机遇大于挑战。未来五年，只要我们凝聚全市人民的智慧和力量，弘扬“十二五”的好经验，坚毅前行，干大事、干实事、干难事、干好事，驰而不息，久久为功，就一定能行稳致远，就一定能让820万邵阳人民进入全面小康，就一定能把邵阳建设成富饶美丽幸福的新邵阳。

“十三五”的指导思想是：高举中国特色社会主义伟大旗帜，坚持“五位一体”发展思路和“四个全面”战略布局，牢固树立创新、协调、绿色、开放、共享的发展理念，按照“三量齐升”的总要求，始终保持“稳中快进”总基调，始终围绕“八个建成”总目标，突出发展、扶贫两大抓手，全力拉长补齐产业发展、新型城镇化、基础设施、县域经济和扶贫五大短板，打造湖南省域副中心、湘中湘西南经济中心、全国区域性交通枢纽，确保全面建成小康社会，让邵阳经济实力稳居全省第二方阵的首位，力争跻身第一方阵。

“十三五”的工作目标是：经济保持中高速增长，增速高于全省平均水平，缩短与全省发展水平的差距。GDP2300亿元，年均增长10%。三次产业结构调整为14:39:47。财政总收入230亿元，年均增长12%。规模工业增加值年均增长12%。固定资产投资年均增长18%。社会消费品零售总额年均增长12%。城镇居民人均可支配收入达到34000元左右，农村居民人均纯收入达到14000元左右，城乡居民人均可支配收入年均增长10%。城镇化率达50%以上。一半以上县域经济达到或超过全省中等水平，其中邵东进入全省十强县。现行标准下农村贫困人口实现整体脱贫，贫困县贫困村全部摘帽，解决区域性整体贫困问题。

1、着力构建经济新结构，打造湘中湘西南经济中心。发挥后发跨越优势，做好“加减乘除”，完成供给侧改革，使邵阳市经济结构在更高水平更大范围实现供需平衡。巩固发展食品加工、装备制造、建材、轻工等优势产业，培育发展生物医药、电子信息、新材料、新能源、节能环保等战略性新兴产业。打造“一核一带多点”工业发展新格局，即宝庆经济技术开发区为核、沪昆高速百里工业走廊为带、县市工业园区为多点，促进工业聚集发展，构建富有竞争力的现代工业供给新结构。围绕农民增收，增强重农固本意识，按照农业生产专业化、标准化、规模化、集约化、产业化的要求，加大农业投入力度，提高农业综合生产能力，培育现代农业经营主体，完善农业服务体系，创建国家级农业科技示范园，农业劳动生产率达到小康标准，加快农业大市向农业强市转变。以打造湘中湘西南现代服务中心为目标，重点建成2—3个湘中湘西南大型综合商贸物流中心，实施“互联网 ”电商行动计划，运用大数据、云计算等信息技术，构建现代物流网、现代金融、现代信息科技等生产性服务产业体系。以建设国内外著名的世界遗产景观和生态文化旅游目的地为目标，构建旅游基础网，壮大旅游支柱产业，把西部打造成以路为径、以景为点的生态文化旅游圈，使之成为张家界—桂林黄金旅游线上的一颗明珠。巩固提升商贸餐饮、居民服务、房地产等生活性服务业，积极推进健康养老、文化创意、电子商务等新兴服务业。

2、坚持精准扶贫、精准脱贫，打赢脱贫攻坚硬仗。把集中连片扶贫和区域协同发展有机结合起来，创新精准扶贫机制和办法，整合扶贫政策和资金，广泛动员和鼓励引导各类企业、社会组织及个人参与扶贫开发，大力实施“六个精准”、“五个一批”，发展生产脱贫50万人，发展教育脱贫2万人，生态补偿脱贫1万人，异地搬迁脱贫8万人，社会保障兜底19万人，确保贫困人口如期脱贫，确保大祥区、双清区、北塔区、邵东县2017年脱贫摘帽，武冈市、城步县2018年脱贫摘帽，隆回县、邵阳县、新宁县、新邵县、绥宁县、洞口县2019年脱贫摘帽。

3、坚定不移推进新型城镇化，建设更加宜居宜业的幸福家园。以以人为本、便民宜居宜业为宗旨，加强城市规划、建设和管理。建设数字城市、智慧城市、紧凑城市和海绵城市，着力解决城市病等突出问题。加快中心城区与邵东、新邵、隆回、邵阳县的融城发展，将东部城镇群建设成为产业兴旺、交通发达、经济繁荣的大城市组团。推动三区与周边县乡区划调整，实施中心城区扩容提质，将核心城区建成100平方公里、常住人口100万的湘中湘西南大城市。建成三环，打通二环，美化一环，织好城市三环网。市中心城区、所有县城、重点乡镇创成国家或省级卫生城市，争创国家森林城市、健康城市、文明城市和交通管理模范城市。增加城市有效投入，20万以上人口县城每年投资在30亿元以上，把县城打造成区域经济中心，增强县城对县域经济的带动和辐射作用，到2020年形成县城人口规模50万人1个、20—30万人5个、10万人左右3个。积极发展重点城镇，打造一批环境优美、特色鲜明的中心镇。推进城市综合行政执法，形成运作市场化、管理网格化、作业精细化、考核标准化的城管体系。

4、继续织实织密“四张网”，提升基础设施共建共享水平。以打造全国区域性交通枢纽为目标，实施铁、公、机互联互通工程，加快构建铁路“三纵三横一连”、高速公路“三纵三横两连”、机场民用航空“一东一西”和通用航空多点的新的交通大格局。提升国省干线路网等级，进一步织密农村公路网络，提升通行能力。推进旅游公路连线环转。积极发展内河水运。加快建设客运、货运枢纽。每万人拥有公共交通车辆达14标台。基本建成小康水利体系，继续推进衡邵干旱走廊综合治理、娄邵盆地基本农田建设和农业综合开发高标准农田建设，完成2处大型灌区和48处中型灌区续建配套与节水改造工程，加快完善市区及9个县城防洪堤闭合体系，继续实施病险水库及水闸除险加固工程，打通防汛抗旱“最后一公里”。建设坚强的能源保障体系，筑牢多层次、高密度、互联互通、稳固安全的电力体系，提升传统能源，继续保持风电全省领先地位，大力推进光伏发电、生物质能、余热（气）余压和地热能、页岩气、农村可再生能源，完善煤、电、油、气供给体系，实现“县县通天然气”。统筹城乡区域信息网，加快“三网融合”，推进4G网络建设。

5、全面深化改革开放，不断增强发展的动力活力。加快转变政府职能，继续简政放权，接稳放准行政审批事项。健全现代财政制度，建立事权和支出相适应的制度。继续深化国企改革，完善国资监管。深化政事、政企、事企改革。落实税制改革任务。实施工业、农业、现代服务业全产业招商，扩大基础设施、社会事业的招商领域和范围，改善投资合作方式。加快鞋业、发制品、打火机等出口基地建设，努力打造国际知名的鞋都、发都、箱包城、食品城。积极推动外经、外贸、外资融合发展。建成邵阳海关、出入境检验检疫机构、公路口岸查验场和邵阳电子口岸，建设海关保税仓库和铁路口岸，实现报关报检便捷化和通关一体化。

6、增进民生福祉，带给群众更多幸福感获得感。继续实行就业优先战略，城镇登记失业率控制在4.5%以内。实现职工医保、居民医保、新农合“三保合一”，推动大病医疗、健康养老及责任险工作，实行低保与社保二合一，完善社会救助和社会福利体系。推进保障性住房建设，基本完成棚户区改造任务，加快住房公积金向城镇自由从业人员和个体工商户延伸。促进妇女儿童全面发展，关爱青少年特别是留守儿童健康成长。提升残疾人服务水平。积极应对人口老龄化。加快推进教育现代化，提升学前、义务教育和高中教育质量，完成“改薄”任务，分期分批通过国家县（市区）域义务教育均衡发展评估验收。有效化解城区大班额。大力发展职业教育，整合职教资源建成市城区职教城，每个县建成1—2所示范性职业学校。提升高校办学水平和科研实验能力。大力推进科技创新，高新技术产业增加值达到400亿元，占GDP比重17%以上。大幅提升科研经费占GDP的比重，每万人发明专利量达到或接近小康标准。加快各县市的体育馆（场）、文化馆、图书馆和文化综合展示馆等文化体育设施建设，人均公共文化财政支出达到200元，有线广播电视入户率超过60%，每万人拥有“三馆一站”公用房屋面积500平方米。健全三级卫生服务网络，进一步落实国家基本药物制度，完善计划生育利益导向政策体系。把绿色化贯穿到新型工业化、城镇化、农业现代化、信息化全过程，做到“五化”互动协调发展。实施“四边五年”绿色行动，森林覆盖率达到64%以上。加强水环境、大气和土壤等污染防治，坚决降低单位GDP化学需氧量、氨氮等主要污染排放量。农村卫生厕所普及率达到75%以上。单位GDP水耗每万元下降到110立方米以下。坚持最严格的耕地保护制度和节约用地制度，单位GDP建设用地占用面积每亿元下降到60公顷。推进矿产资源综合利用示范基地和绿色矿山建设。坚决防止重特大生产安全事故。每万人拥有律师3人。大力建设平安邵阳、法治邵阳、文明邵阳和诚信邵阳，提升社会管理水平。

三、认真做好2016年工作

经济社会发展预期目标是：GDP增长10%左右，规模工业增加值增长12%，固定资产投资增长18%，社会消费品零售总额增长12%，财政总收入增长12%左右，利用外资增长10%，利用内资增长20%，进出口总额增长15%，城乡居民人均可支配收入增长10.5%，常住人口城镇化率达到45%，人口自然增长率控制在9‰以内，居民消费价格涨幅控制在3%左右，万元GDP能耗下降3%。

实施重点项目300个，概算总投资3200亿元，完成年度投资600亿元以上，推进重大前期项目150个，完善招商项目库200个，力促项目早获批、早开工、早投产。重点抓好十个方面的工作：

1、推进供给侧结构性改革,加快工业转型升级

推进园区建设和管理。以更大力度推进湘商产业园建设，新开工标准厂房170万平方米，续建130万平方米，入园企业390家。尽快整合宝庆工业集中区和邵阳经开区，申报国家级经济技术开发区和国家级创业示范基地，力争实现工业总产值260亿元，税收20亿元。加快各类农业科技示范园和雀塘湘中湘西南循环产业园区建设。推进大众创业、万众创新，充分利用各类园区、基地和高校、职校、企业，在新兴制造业和现代服务业等领域，构建众创空间示范基地和创业创新聚集区。

推进传统产业改造和新兴动能驱动。贯彻落实《中国制造2025》，支持三一湖汽、恒天九五、湘窖酒业等龙头企业转换动能。促成亚洲富士电梯早日投产，加快统一饮料、百威啤酒等大型食品企业建设。壮大以湘中制药为重点的生物医药业。持续抓好“四百工程”。轻工、建材、食品和农产品加工等传统产业增长15%以上。战略性新兴产业中的规模工业企业产值占全部规模工业企业产值20%以上。

推进降低成本为实体经济减负。加快企业服务平台建设，积极对接并用好中央打出的降低企业税费、社会保险费、财务成本、电力价格和物流成本等“组合拳”。落实“一行一策、一厂一策”等精准帮扶措施，切实帮助企业排忧解难。用活人才存量，扩大人才增量，对重点企业实施“一企一干部一专家”行动。

2、提高农业供给体系质量和效率，大力推进农业现代化

加强农业产业基地建设。按照藏粮于地、藏粮于技的要求，确保粮播面积910万亩、总产340万吨以上，蔬菜种植面积稳定在150万亩以上。开展水稻、玉米、油菜等高产创建活动，推进油茶、茶叶、奶业、金银花、中药材、龙牙百合等特色农产品产业化、规模化、品牌化、信息化。种植烤烟12万亩，收购20万担。出栏牲猪1300万头，创建畜禽渔标准化健康养殖示范场30个，确保主要畜禽和水产品产值稳定增长。

发展农副产品加工。实施农副产品加工推进计划，落实农业产业化龙头企业扶持政策，重点扶持10家骨干龙头企业，加快农副产品加工企业集群发展。走产学研、农科教相结合的发展路子，加大高新技术的引进和科技成果的推广应用力度，研究开发一批附加值高、特色明显、市场广阔的高新产品。

壮大农业经营主体。推动建立农村土地流转服务中心，促进耕地向专业大户、家庭农场、龙头企业流转。实施农业合作社行动计划，争取每个乡镇有1—2个农业综合合作总社、每个村有1—2个农业专业合作社或分社。发展农机经营实体，推广先进适用农机具3万台，农机总动力达到490万千瓦，水稻生产耕、种、收全程机械化水平达到66%。完善农产品质量安全监管体系，创建农产品质量安全监管示范单位。

3、加快发展现代服务业，创造新供给，引导新需求

做大做强物流业实体。在铁路沿线布局8—10平方公里现代物流产业园。开工建设怀邵衡东货场湘中湘西南现代生产性物流中心。大力培育商贸物流重点企业，积极引进大型知名商贸企业、物流公司、快递公司。不断完善便民惠民的市场流通体系，继续推进城乡农贸市场标准化改造，彻底取缔马路市场。大力发展“互联网 ”电子商务，促进线上线下融合发展，筹建邵阳电子商务智慧产业园。

壮大旅游业。培育旅游“六要素”实体经济，大胆放开引进民间资本投资旅游。实施旅游景区精品战略，开发、包装、建设崀山、南山、黄桑等10个重点景区，启动市城区旅游景点建设三年行动，规划建设一流的大型综合游乐场。针对重点区域和重点人群实施精准营销，写好“旅游 文化、旅游 体育、旅游 养生”等旅游促销品牌文章。贯通崀山与桂林、张家界高速公路通道，共享三地客源市场，形成三地旅游“一旅通”。接待国内外游客3200万人次，实现旅游总收入240亿元。

引导金融服务实体经济。探索建立银企合作新机制，金融机构扩大信贷投放规模，降低贷款门槛，破解抵押瓶颈，有序化解“坏账”，发展新型金融产品，提高金融服务水平。引导企业和项目通过多种方式融资，新增各类融资200亿元以上。金融机构存款余额增长16%以上，贷款余额增长18%以上。保费收入增长15%。抢抓注册制改革机遇，精准推进企业在主板、新三板、新四板、高新板等上市融资，摆脱信贷依赖症。继续创建金融生态区，加强民间融资管理，牢牢守住不发生区域性金融风险的底线。

激活房地产消费。推进户籍制度改革，加快农民工市民化，政府政策激励，企业适当降价，化解房地产库存，打通房产供需通道。盘活城区公租房存量，逐步解决下岗职工、城镇困难群众、农民工住房问题。

4、以建档立卡对象脱贫为目标，着力提升扶贫的精准度

明确年度扶贫任务。按照“两不愁、三保障”和“六到位、六落实”的要求，年内减少贫困人口21万。发展生产脱贫11.7万人，发展教育脱贫0.62万人，生态补偿脱贫0.3万人，异地搬迁脱贫2万人，社会保障兜底6.5万人。

创新扶贫方式方法。围绕“五个一批”任务，积极对接国家政策，主动争取扶贫指标，落实多种扶贫措施。推广光伏扶贫、旅游扶贫、电商扶贫和资产收益扶贫等方式，促进贫困地区村村有产业、贫困家庭户户有家业。支持贫困地区“两后生”参与大中专学历教育和职业教育，帮助3000个贫困家庭学生参加“雨露计划”培训并推荐就业。培训农民3万人次，使贫困家庭劳动力人均掌握1项以上种养加实用技术。年内搬迁6464户。用好生态补偿资金，将重点生态功能区部分困难群众转成护林员、护水员、护路员、保洁员等，实行就地就能就业。确保市县财政对低保对象的兜底。

动员全社会扶贫。把扶贫工作扛在肩上、抓在手上，掌握主动权，推广武冈危房改造经验，整合各类涉农资金，促进政策、资金、人才等向贫困地区和贫困对象倾斜。用好用活金融扶贫政策。广泛动员社会力量参与驻村结对帮扶，努力构建“大扶贫”格局。

5、准确把握后发优势和发展空间，把城镇化建设作为新的经济增长点

加强城镇规划。不断创新规划理念和方法，增强规划的科学性、指导性，加强控制性详细规划的公开性和强制性。加强对城市的空间立体性、平面协调性、风貌整体性、文脉延续性等方面的规划和管控。统筹各类空间性规划，推进“多规合一”。各县城和重点镇要完成编制或修改城市地下管线综合规划、地下空间利用规划，以及消防、给水、排水、电力、燃气等基础设施专项规划。

建设宜业宜居的中心城市。完成资江北路、双清路、宝庆西路延伸段等道路改造。推进龙须塘片区“两沟两路”和白马大道、北塔大道北段建设，确保新华南路二期（含隧道）、建设北路（含隧道）、魏源西路、雪峰北路、雪峰南路、雪峰大桥、桂花大桥建成通车。完成三环路前期工作，争取年底全线开工。实质性开工建设资江南岸风光带、北塔公园、东塔公园和邵水两岸风光带。推进桂花渡水厂、邵水桥老桥改造。加快停车场、综合管廊等配套设施建设，完善城市整体功能。用市场化理念推进城中村改造。坚决清理处置批而未供和闲置土地。

加快县城和重点镇建设。重点抓好9个县（市）城和廉桥、高沙、六都寨、武阳等中心（示范）镇扩容提质，加快供排水、垃圾处理等配套设施建设，强化城镇管理。

提升城市管理水平。扎实推进城市综合管理改革，逐步实现城管网格化、规范化、制度化。国家卫生城市通过暗访验收。按照“序化、净化、绿化、亮化、美化”要求，持续开展市容秩序、户外广告、门店招牌、渣土运输、工地扬尘、管线、违法建设、交通等专项整治。

6、着力补短板，进一步改善基础设施软硬条件

推进交通建设。完成征地拆迁，全力配合怀邵衡铁路建设。积极争取邵永铁路扩改进入可研，继续争取兴永郴赣铁路和呼和浩特至南宁高铁经邵阳纳入国家“十三五”建设规划，开工建设邵阳南客专高铁站。启动建设邵阳东1000万吨货运站。武靖高速完成主体工程，开工白新高速，安邵、新桂高速竣工通车，积极做好呼北高速邵阳段、新宁至永州、城步至龙胜、洞口至溆浦高速公路的前期工作。确保5条干线公路共118公里建成通车，加快12条共246公里干线公路续建项目建设。确保武冈机场年底试飞，启动邵东军民两用机场和多个通用机场的前期工作。加快坪上综合客运枢纽建设，正式启动市区邵阳汽车南站综合客运枢纽和东站超大型汽车站建设。新建邵东汽车东站、新宁崀山汽车站、邵阳县峡山客运站、隆回汽车北站等客运站。抓好邵阳港建设，推进资江干流航道整治。

推进水利建设。争取衡邵干旱走廊综合治理项目提上省里议事日程，推进犬木塘、木瓜山水库建设前期工作。利用水务投平台，启动市区桂花桥至西湖桥、县城及其乡镇的防洪堤建设。做好秀山、威溪、张家冲等7座大中型水库除险加固前期工作，完成鸟竹园、云水铺、田心坝等工程年度建设任务，推进封江坝等15座大中型水闸前期工作。竣工验收4个抗旱应急水源工程。加强小型农田水利基础设施建设和管理。

推进能源建设。完成农村未改村电网改造任务，重点加强市城区和县城的电网提质改造。继续抓好风电、光伏等新型能源开发建设，支持苏宝顶风电二期、毓兰光伏等项目规模化、集群化发展。推进“气化邵阳”，加快输气管道建设。建成CNG加气站1座，新建燃气中压主干管网60公里，改造老旧燃气管网80公里。市城区完成“煤改气”。

7、持续深化改革开放

推进关键领域改革。完成市县政府机构改革、乡镇村区划调整和党政机关公务用车改革。加快财税体制改革，完善预算管理机制，规范国库集中支付，更大范围、更大力度扩大政府购买服务。启动政企、事企改革二年行动。加强政府服务平台建设，推行“两集中、两到位”工作。建成全市资源交易服务中心。开展不动产统一登记。推进供销合作社系统综合改革。深化农村金融改革，完成10家农商银行、9家村镇银行的开业和批筹工作，推进农村土地承包经营权抵押贷款。做好国有林场改革扫尾工作。

扩大外经贸规模。重点扶持年进出口额超1000万美元企业，争取培育1—2家进出口超亿美元企业。支持出口企业加快“走出去”，争取新增境外投资企业20家，新申请马德里国际注册商标10件以上。开工建设邵阳海关、检验检疫机构和商品查验场所。

提升招商引资实效。按照“引老乡回故乡建家乡”、“带技术带市场带项目”、“产品有市场、企业有利润、员工有收入、政府有税收”的“三乡三带四有”要求，宽领域多行业广泛招商。完成1亿元以上招商项目大县市20—50个，其他县区10—20个，其中工业投资项目占60%以上。建立健全签约项目推进落地、跟踪调度、履约考核机制，力促湘台大会、湘商大会等招商活动签约项目落地。

8、更加关注民生，有效增加公共产品和公共服务供给

努力促进更多就业。继续实施“创业引领计划”、“创业扶持计划”。新增城镇就业4.9万人，失业人员再就业2.7万人，各类就业培训4.5万人，农村劳动力转移就业稳定在130万人以上。

提升社会保障水平。进一步抓好各类社会保险扩面和基金征缴，落实机关事业单位养老保险、城乡居民大病保险和住房公积金改革等各项惠民政策，发展企业年金等补充性养老保险。启动敬老院、福利院对服务对象全覆盖行动。推动以“两房两棚”为重点的保障性安居工程，新增公租房18670套，改造农村危房45000户、城市棚户区49118户、国有工矿棚户区956户。实施货币化安置50%以上，完成乡镇教师、医生周转房建设。

促进教育协调均衡发展。提高学前三年入园率、九年义务教育巩固率和高中阶段入学率，完成18所农村公办幼儿园、180所合格学校建设任务。继续实施城区化解大班额三年行动计划，开工建设一中搬迁项目，加快7所重点学校建设。支持发展高等教育、职业教育和民办教育、特殊教育，整合城区中职学校资源，启动职教城建设五年行动计划。确保邵阳幼师高等专科学校通过省里评估验收。解决进城务工人员子女接受义务教育问题，高度关爱农村留守儿童，高度重视校园、校车安全。

加快发展文化体育事业。市文化艺术中心的“五馆二中心一剧院”正式对外开放，市体育中心的主场馆主体竣工。加快公共文化服务体系建设，完善市、县、乡、村四级文化设施网络。“三馆一站”免费开放。继续推进广播电视“户户通”、“村村响”和无线覆盖提质、农村公益电影放映等惠民工程，启动城镇电视电信网络工程。加大重点文物和非物质文化遗产保护力度，推动绥宁侗寨申世遗工作，修复宗祠古建筑群。加强“扫黄打非”，规范文化市场秩序。广泛开展全民健身运动，促进群众体育和竞技体育全面发展，举办第三届市运会。支持城步苗族自治县成立60周年县庆。按时完成乡镇（街道）简志，全面推进第二轮修志工作。

支持科技研发。鼓励企业科技创新，研发经费支出占GDP比重提高0.25个百分点，发明专利申请量、拥有量和专利总量增长12%，新增高新技术企业60家，宝工区升为省级高新区。

推进医疗卫生事业建设。开展村卫生室清零行动，全面完成村级卫生室建设任务。配足配强县乡村医疗人员，完善县乡村医疗机构人员、编制、管理体制。全面推进分级诊疗体系和重大疾病防治设施建设。支持民营医院发展和重点医疗机构改扩建，完成市中心医院东院主体工程，启动市第二人民医院北扩工程和中医院肿瘤大楼工程。稳妥实施全面两孩政策，严格控制政策外多孩生育，计划生育工作进入全省优良行列，做好母婴健康安全和出生缺陷干预工作，促进人口健康均衡发展。

9、改善环境质量，提高绿色化和生态文明水平

启动四边五年绿色行动。重点推进路、水、城、村边万里绿色带建设，开展城区、乡镇、村庄增绿行动。制定秀美村庄标准，各县市区20%的村建成秀美村庄。新建2个森林公园、2个专业花卉园和1个农林科技示范园。实施人工造林36万亩，低效林改造15.5万亩、封山育林32万亩、中幼林抚育54万亩。每个县市区在城郊建成1—2个农村生态绿色美丽富裕示范村。

坚决整治环境污染。对直接影响环境质量的扬尘、噪音、汽车尾气等污染问题进行集中整治，启动26个大气污染防治项目。不断提高城区空气质量的优良率，确保空气优良天数超过260天。强化对沿江、沿河企业环境污染隐患的排查整治，加快黑臭水体治理，坚决堵住资江、邵水直排，确保资江和邵水等重点流域的饮水安全。推进洋溪桥污水处理厂改扩建工程，建成市污泥集中处置中心和北塔污水处理厂，实现污泥无害化处理率70%。加强龙须塘化工区环境综合治理，限期整治或依法关停违法排污企业。加快雀塘湘中湘西南循环产业园建设。支持邵东、新邵、武冈、新宁等重点区域开展重金属污染防治，综合治理邵东石膏矿采空区和采煤沉陷区。推进污染减排工程项目，严格控制主要污染物排放新增量。突出结构性减排，坚决淘汰落后产能。

持续推进生态创建。抓好以生态县、环境优美乡镇、秀美村庄为载体的生态建设与保护工作，搞好生态文明示范工程和城步国家公园体制试点。继续深化农村环境综合整治，开展“三清五改”，完成646个人居环境重点村整治，形成县、乡、村环境清洁体制机制，着力解决危害群众身体健康的环境污染问题。推广应用农村清洁能源、废弃物综合利用和污染物无害化处理技术。推进农村畜禽养殖污染集中整治，控制面源污染。启动农村集中饮用水水源划定和保护工作。加强国家湿地公园的保护和建设，创建全国水生态文明城市。

10、夯实基层基础，坚决维护社会和谐稳定

筑牢安全生产防线。深入推进安全监管防控体系建设，高危企业标准化建设达标率100%，网格化管理全覆盖。加大重点行业领域安全隐患排查治理力度，严厉打非治违，严格责任追究，坚决遏制重特大事故发生。继续推进落后小煤矿和烟花爆竹生产企业的关闭退出，推进非煤矿山整顿关闭，不再新批年生产能力10万吨以下的非煤矿山。进一步加强地质灾害防治体系和食品药品安全社会共治体系建设。

扎实推进平安邵阳建设。畅通“12345”政府服务热线，提升热线解决群众诉求的能力。落实依法逐级走访，全面推行“网上信访”，继续实行领导包案化解信访积案，加强县乡矛盾纠纷调处中心规范化建设，构建多元化社会矛盾化解机制。开展市县两级公安、消防大建设三年行动，逐步缩小与发达地区的差距。坚持命案必破、黑恶必除，依法严厉打击危害人民群众生命财产安全的违法犯罪活动。创新社会治安防控体系，健全群防群治队伍，推进视频监控工程、社会治理信息平台建设，重点整治社会治安混乱的地区和场所，彻底改变社会治安综合治理落后状况。加强网络信息安全管理，打击网络违法犯罪。完善戒毒软硬设施和场所，坚决打击毒品犯罪。认真开展社区矫正和安置帮教工作，创建无传销城市。

突出基层队伍建设。加强以基层党支部建设为核心的基层组织建设，尤其是加强基层队伍建设。制定城镇社区、农村村部硬件建设标准，切实解决社区、村部工作场所和设施落后、不齐的问题。人力、物力、财力向社区、村倾斜，配强配足人员，县市区、乡镇街道干部下沉，充分发挥社区、村第一支部书记的作用，继续强化交通、信访、治安、安全四支队伍建设，加大财政对社区、村工作经费和报酬的投入，做到有人愿意做事、有钱能够办成事，做到美好社区、村全覆盖。

各位代表，我们将继续加强国防动员、国防教育和人民武装工作，支持驻邵部队建设，促进军地融合协调发展。加强人民防空、国家安全工作，加快新型疏散基地建设。做好双拥工作，争创全国双拥模范城。认真落实军转安置任务。

各位代表，做好“十三五”及2016年各项工作，关键在人，在“关键少数”。我们将始终把依法行政摆在首位。强化法无授权不可为，治理乱作为，努力建设法治政府。我们将始终把责任扛在肩上。强化法定职责必须为，治理不作为，克服“庸懒散”。我们将始终把人民群众放在心中。依靠群众，关心群众，服务群众，把群众的问题当作工作方向和目标。我们将始终把接受监督置于全程。依法接受人大及其常委会的法律监督和工作监督，自觉接受政协的民主监督，虚心接受各民主党派、工商联、无党派人士和人民团体的监督，主动接受媒体和公众监督。我们将始终把纪律规矩挺在前面。坚持“三严三实”，弛而不息纠正“四风”，牢固树立过紧日子的思想，全面公开政府及各部门预决算和“三公”经费，实现审计全覆盖。坚持有腐必反、有贪必肃，对各种腐败行为决不姑息，树立政府为民务实清廉的良好形象。

各位代表，风帆启航再驰骋，大海扬波作和声。带领820万各族人民奔小康是我们这一代人的使命担当，一场长达五年的“赶考”、“大考”，只能考好，没有退路。让我们在中共邵阳市委的坚强领导下，登高望远，脚踏实地，卧薪尝胆，砥砺前行，为夺取全面建成小康邵阳的伟大胜利而努力奋斗！
